# Serial Metabolic Evaluation of Perihematomal Tissues in the Intracerebral Hemorrhage Pig Model

**DOI:** 10.3389/fnins.2019.00888

**Published:** 2019-08-21

**Authors:** Muhammad E. Haque, Refaat E. Gabr, Sarah D. George, Xiurong Zhao, Seth B. Boren, Xu Zhang, Shun-Ming Ting, Gunghua Sun, Khader M. Hasan, Sean Savitz, Jaroslaw Aronowski

**Affiliations:** ^1^Institute for Stroke and Cerebrovascular Diseases, McGovern Medical School, The University of Texas Health Science Center at Houston, Houston, TX, United States; ^2^Diagnostic and Interventional Imaging, McGovern Medical School, The University of Texas Health Science Center at Houston, Houston, TX, United States; ^3^Biostatistics, Epidemiology, and Research Design Component, Center for Clinical and Translational Sciences, McGovern Medical School, The University of Texas Health Science Center at Houston, Houston, TX, United States

**Keywords:** intracerebral hemorrhage, pig ICH model, perihematomal edema, magnetic resonance spectroscopy, serial neuroimaging study

## Abstract

**Purpose:**

Perihematomal edema (PHE) occurs in patients with intracerebral hemorrhage (ICH) and is often used as surrogate of secondary brain injury. PHE resolves over time, but little is known about the functional integrity of the tissues that recover from edema. In a pig ICH model, we aimed to assess metabolic integrity of perihematoma tissues by using non-invasive magnetic resonance spectroscopy (MRS).

**Materials and Methods:**

Fourteen male Yorkshire pigs with an average age of 8 weeks were intracerebrally injected with autologous blood to produce ICH. Proton MRS data were obtained at 1, 7, and 14 days after ICH using a whole-body 3.0T MRI system. Point-resolved spectroscopy (PRESS)-localized 2D chemical shift imaging (CSI) was acquired. The concentration of *N*-Acetylaspartate (NAA), Choline (Cho), and Creatine (Cr) were measured within the area of PHE, tissues adjacent to the injury with no or negligible edema (ATNE), and contralesional brain tissue. A linear mixed model was used to analyze the evolution of metabolites in perihematomal tissues, with *p*-value < 0.05 indicating statistical significance.

**Results:**

The perihematoma volume gradually decreased from 2.38 ± 1.23 ml to 0.41 ± 0.780 ml (*p* < 0.001) over 2 weeks. Significant (*p <* 0.001) reductions in NAA, Cr, and Cho concentrations were found in the PHE and ATNE regions compared to the contralesional hemisphere at day 1 and 7 after ICH. All three metabolites were significantly (*p* < 0.001) restored in the PHE tissue on day 14, but remained persistently low in the ATNE area, and unaltered in the contralesional voxel.

**Conclusion:**

This study highlights the potential of MRS to probe salvageable tissues within the perihematoma in the sub-acute phase of ICH. Altered metabolites within the PHE and ATNE regions in addition to edema and hematoma volumes were explored as possible markers for tissue recovery. Perihematomal tissue with PHE demonstrated a more reversible injury compared to the tissue adjacent to the injury without edema, suggesting a potentially beneficial role of edema.

## Introduction

Intracerebral hemorrhage (ICH) often results in hemiparesis or hemiplegia. ICH accounts for about 10–15% of all strokes, and is associated with a high mortality and long-term disability rate (Feigin et al., 2009). ICH-induced deaths may occur within hours to days of the ictus (Feigin et al., 2009; Hemphill et al., 2015). The sequelae of ICH pathogenesis consist of primary injury with the rupture of blood vessels, physical compression, and disruption of brain tissues by the expanding blood mass. The secondary injury is associated with hematoma toxicity, oxidative stress, neuroinflammation, blood-brain barrier disruption, and consequent robust edema Perihematomal edema (PHE) (Wagner et al., 2003; Cherubini et al., 2005; Babu et al., 2012; Selim and Sheth, 2015; Lim-Hing and Rincon, 2017; Bernstein et al., 2018).

Initial cytotoxic edema progresses into mainly vasogenic edema, which is coupled with extravasation of blood fluid that contains plasma proteins, and has been proposed to contribute to additional injury (Xi et al., 2001; Keep et al., 2012a; Selim and Norton, 2018). One study reported a significant proportion of swollen cells in the inner and outer rims of the perihematoma with a small proportion of shrunken dark cells within 24 h after onset (Qureshi et al., 2001). ICH-induced neuroinflammation has been characterized by the presence of neutrophils, microglia, and macrophage/monocyte around the hemorrhagic foci (Castejon et al., 2005; Bakhshayesh et al., 2014; Zhao et al., 2007, 2017; Zhang et al., 2017). The location, expansion and retraction of hematoma and PHE volumes are used as a prognostic tool in humans and rodents (Aronowski and Hall, 2005; Falcone et al., 2013; Grunwald et al., 2017; Al-Shahi Salman et al., 2018; Klahr et al., 2018; Volbers et al., 2018). Because of the clinical importance of PHE, there is great interest to understand the pathophysiology and develop therapeutic agents targeting PHE. Serial and cross-sectional neuroimaging studies have reported a reduction of edema over time (Murthy et al., 2016; Ozdinc et al., 2016; Rodriguez-Luna et al., 2016; Liu et al., 2019), however, there is little known about the functional and structural restoration of those tissues after edema resolution.

*N*-acetylaspartate (NAA), choline (Cho), and creatine (Cr) are three major brain metabolites, seen on proton magnetic resonance spectroscopy (MRS) (^1^H-MRS), which are often used as surrogate markers of neuronal health, cell proliferation, and cellular energy, respectively (Prado et al., 2002; Madhavarao and Namboodiri, 2006; Rigotti et al., 2007; Rackayova et al., 2017). Alterations in these metabolites were documented in patients with ischemic stroke, providing useful markers of tissue health (Fenstermacher and Narayana, 1990; Felber et al., 1992; Kang et al., 2000; Bivard et al., 2014). However, only a limited number of reports characterized these changes in patients with ICH (Kobayashi et al., 2001). Wagner reported a significant reduction in NAA in patients with a subarachnoid hemorrhage and Kobayashi reported a reduction in the ipsilesional NAA/Cr ratio in the motor cortex in patients with poor outcomes (Kobayashi et al., 2001; Wagner et al., 2013). Another study also demonstrated a significant reduction in NAA/Cr ratio in the primary motor cortices remote from the locus of injury (Yang et al., 2017). However, only few neuroimaging studies investigated metabolic changes in the PHE. Two studies by Carhuapoma et al. (2000, 2005) reported the presence of lactate and increased apparent diffusion coefficient in the PHE regions in ICH patients. In an animal study, Yin et al. (2006) showed significant apoptosis and reduction in NAA/Cr ratio in the PHE within 24 h of ICH onset. However, to the best of our knowledge, no study used spectroscopy to probe metabolic activities of the perihematoma region during post-ICH recovery, especially to understand the fate of peri-hematomal tissue regarding its functional recovery vs. irreversible damage.

The primary objective of this study was to assess the metabolic integrity of the perihematoma region over 2 weeks after injury using the ICH pig model. We used non-invasive MRS to probe neuronal viability/functionality in the perihematoma and the tissue adjacent to the injury with no apparent edema.

## Materials and Methods

Animal studies were approved by our institution’s Animal Welfare Committee. The studies followed the guidelines outlined in the *Guide for the Care and Use of Laboratory Animals* from the National Institutes of Health.

### Induction of ICH

Fourteen male Yorkshire pigs were studied with average age/weight of 8 ± 2 weeks/13.4 ± 2.4 kg. Procedural details were described elsewhere (Haque et al., 2018). Briefly, animals were sedated with ketamine (25 mg/kg, IM) and maintained under isoflurane throughout surgery. During the surgery, animals were wrapped in a blanket (to maintain body temperature), intubated, and catheterized to monitor physiological variables. To produce lobar ICH, a cranial burr hole was drilled 11 mm to the right of the sagittal and 11 mm anterior to the coronal suture. Hemorrhage was induced by pressure controlled infusion of autologous blood into the right frontal hemisphere, first 1.0 mL infused over 10 min followed by additional 1.5 ml after a 5 min interval.

### Image Acquisition

Pigs were serially imaged on 1, 7, and 14 days after the surgery. During imaging, animals were ventilated with an average of 18 breaths-per-minute with maximum airway pressure of 20 cm-H_2_O, oxygen saturation level (SpO_2_) of 97%, typical end-tidal CO_2_ of 47 mmHg, heart rate 120 beats-per-minute, and body temperature 98.6°C. Anesthesia was maintained with 2% isoflurane mixed with oxygen.

Animals were positioned prone in 3.0 Tesla Philips Ingenia MRI system (Philips, Best, Netherlands) and imaged using an eight-channel head coil. Anatomical imaging included: 3D T_1_-weighted (TR/TE = 10/4.8 ms), 3D coronal T_2_-weighted (TR/TE = 3500/267 ms), and 3D fluid attenuated inversion recovery (FLAIR, TR/TI/TE = 4800/1650/129 ms) with a field-of-view of 200 × 200 × 86 mm^3^. Multivoxel spectral data were obtained using point resolved spectroscopy (PRESS)-localized two-dimensional chemical shift imaging (CSI) with TR/TE = 2000/144 ms, matrix size = 9 × 9, bandwidth = 2000 Hz, and voxel size = 15 × 15 × 15 mm^3^.

### Spectral Analysis and Metabolite Quantification

All spectral integration and phase correction were performed manually using in-house software in Matlab (The Mathworks, Natick, MA, United States). The metabolite peaks at 2.01, 3.03, 3.19 ppm were referenced to NAA, Cr, and Cho, respectively, with respect to the unsuppressed water signal at 4.7 ppm. The absolute concentration of the metabolites was quantified using the unsuppressed water peak as an internal reference (Ernst et al., 1993; Kreis et al., 1993) with T_1_ and T_2_ correction for metabolite and water using the following equation:

$$C_{m}=\frac{A_{m}\,N_{w}\,\left(1-e^{-\mathrm{𝑇𝑅}/T_{1\,w}}\right)\,e^{-\mathrm{𝑇𝐸}/T_{2\,w}}}{A_{w}\,N_{m}\,\left(1-e^{-\mathrm{𝑇𝑅}/T_{1\,m}}\right)\,e^{-\mathrm{𝑇𝐸}/T_{2\,m}}}\,C_{w}\;\;\;\;\;\;\;\;\;\;\;\;\;\;\;\;\;\;\;\;\;(1)$$

where *C*_*m*_ is metabolite concentration, *A*_*m*_ (*A*_*w*_) is the area under the metabolite (water) peak, T_1m_ (T_1w_) is the longitudinal relaxation time of metabolite (water), T_2m_ (T_2w_) is the transverse relaxation time of metabolite (water), *N*_*m*_ (*N*_*w*_) is the number of hydrogen atoms in the metabolite (water) molecule, TR is the repetition time, TE is echo time, and *C*_*w*_ is the reference water concentration in tissue. Previously reported T_1m_, T_1w_, T_2m,_ T_2w,_ and *C*_*w*_ at 3T systems were used in the calculation (Reyngoudt et al., 2010). Concentration is reported in milli-mole (mM) units.

To characterize biochemical events in brain tissue directly affected by ICH, we identified one voxel within the PHE, ATNE, and contralesional hemisphere and measured its absolute metabolite concentrations.

### Lesion Volume Measurements

A semi-automated seed growing algorithm in Analyze 12.0 (Analyze Direct, Inc., KS, United States) was used to measure edema and hematoma volume on FLAIR images by a single rater. The rater selected two seed points within the hyperintense edema and hypointense hematoma and a region-growing algorithm automatically expanded the seed points within the 3D space of the image. Manual editing of the lesion volume was done when necessary.

### Statistical Analysis

Cerebral metabolite concentrations were analyzed by the mixed model (Littell et al., 1998). The fixed effects in the model included brain regions (PHE, PHNE and contralesional), polychotomous time (1, 7, and 14 days), and interaction between hemisphere and polychotomous time. The random effects in the model included animal and interaction between animal and brain regions. These random effects led to a nested covariance matrix accounting for correlation of measurements due to the same animal and a different level of correlation for measurements due to the same hemisphere of the brain. The edema and hematoma volume were analyzed by the mixed models including polychotomous time as a fixed effect and animal as a random effect. Two-sided *p-*values of less than 0.05 were considered statistically significant. All statistical analyses were performed using the SAS software (version 9.4, the SAS Institute, Cary, NC, United States).

## Results

Twelve pigs completed all three imaging time points. One pig died during imaging on day 1 and was excluded from the analysis. Another pig died one day prior to the last imaging on day 14.

**FIGURE 1:**
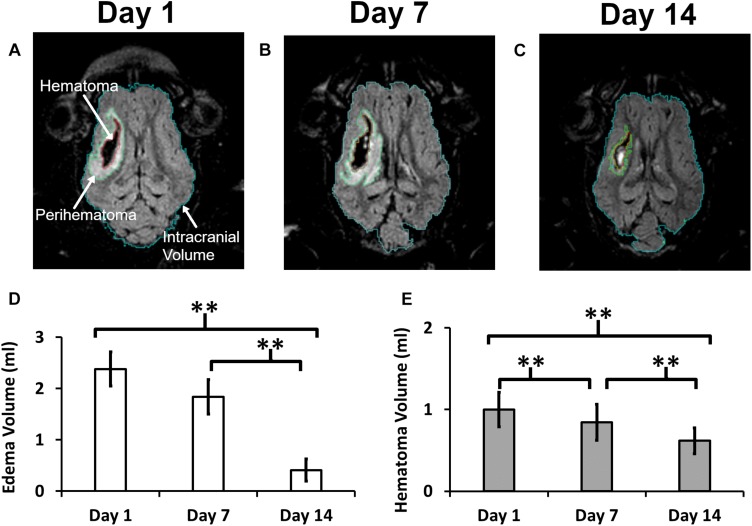
A quantitative summary of the change in hematoma and edema volume in a post-ICH pig brain. Panels **(A–C)** illustrate a representative MRI image showing outlined boundaries of intracranial volume, perihematoma, and hematoma at days 1, 7, and 14, respectively. Panels **(D,E)** show Perihematoma edema **(D)** and hematoma **(E)** volumes. A double asterisk (^∗∗^) denotes the significant temporal ipsilesional difference between the time point (*p* < 0.05). The error bars represent standard error in the mean.

Representative MRI images demonstrating changes in the PHE region and hematoma volumes at three time points in a representative pig are shown in Figure 1. The computer calculated region of interest (ROIs) showing the boundaries were used to calculate the intracranial (ICV), hematoma, and edema volumes. There was no statistically significant change in ICV between day 1 (70.3 ± 6.5 ml), day 7 (72.9 ± 7.6 ml), and day 14 (74.2 ± 7.6 ml). ICH resulted in a 2.38 ± 1.23 ml PHE on day 1 that progressively resolved over the course of 2 weeks, reducing by 23.1% on day 7 and by 82.7% on day 14 (*p* < 0.001) (Figure 1D). Our model of ICH generated a hematoma volume of 1.00 ± 0.79 ml on day 1 after the surgery. The hematoma underwent progressive clearance and was reduced by 15.7% on day 7 and by 38.3% on day 14 (*p <* 0.001), as compared to day 1 (Figure 1E), indicating effective hematoma clearance.

**FIGURE 2:**
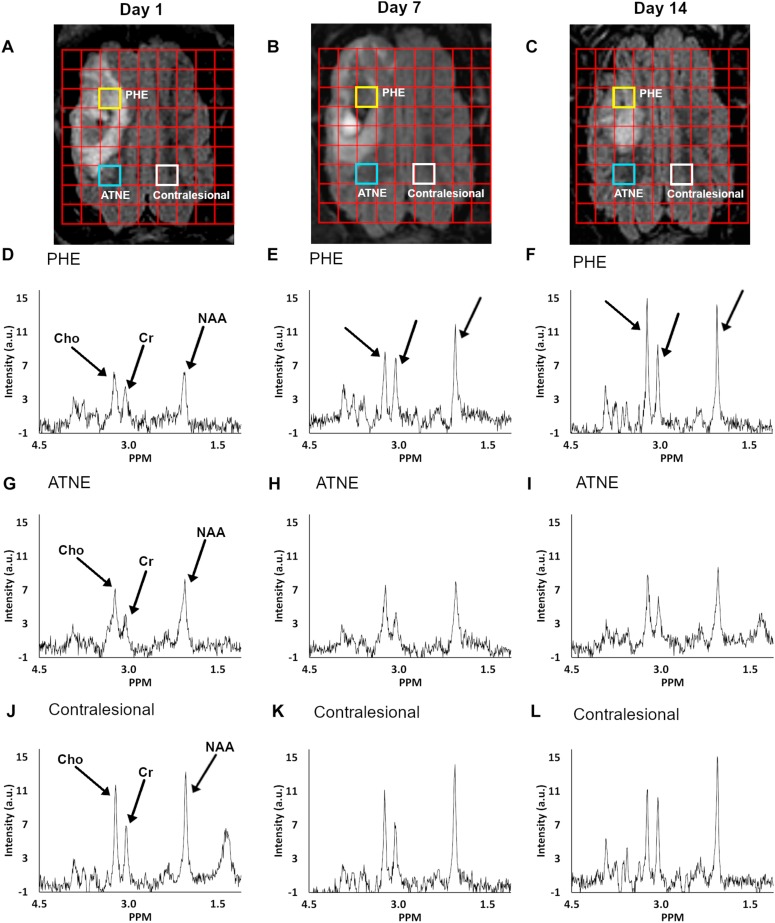
Serial FLAIR MRI images overlaid with a multi-voxel grid of corresponding temporal spectra with highlighted voxels of interest **(A–C)**. Panels **(A–C)** show the PHE, ATNE, and contralesional voxel location at day 1, 7, and 14, respectively, from which the metabolite spectra were measured. MR spectroscopy graphs at days 1, 7, and 14 (columns) are shown for voxels from the PHE **(D–F)**, ATNE **(G–I)**, and the contralesional regions **(J–L)**.

**FIGURE 3:**
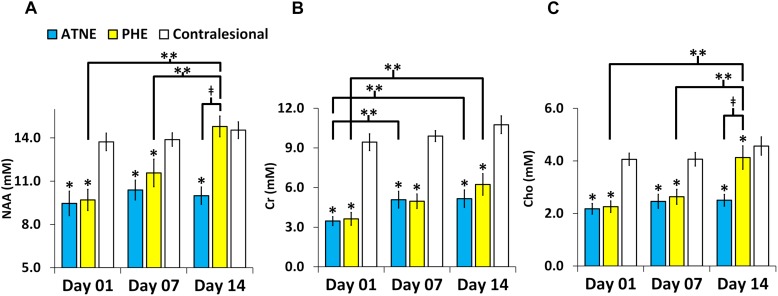
Metabolite concentration in the perihematomal edema (PHE), adjacent tissues with no edema (ATNE), and contralesional voxels over two week follow-up post ICH. **(A–C)** Represent serial NAA, Cr, and Cho concentrations, respectively. The asterisk (^∗^) denotes significant (*p* < 0.001) hemispheric differences, the double asterisk (^∗∗^) denotes significant (*p* < 0.001) temporal difference and (‡) denotes significant (*p* < 0.001) change between PHE and ATNE. The error bars represent standard error in the mean.

Representative anatomical locations of MRS data collection are illustrated in Figures 2A–C. The locations were similarly applied across all 13 analyzed pigs and were selected on day 1 to signify presence of PHE, adjacent non-edematous tissues (ATNE), and the contralesional brain tissue. Our analyses demonstrate a clear pattern of metabolite loss around the hematoma at day 1 after ICH with strong restoration of NAA and Cho concentrations in the PHE, and unexpectedly poor restoration of all the metabolites in ATNE tissue, as assessed on day 7 and day 14 (Figures 2D–L). Specifically, the NAA concentration in both PHE and ATNE regions was reduced (*p* < 0.001) in response to ICH, as compared to contralesional tissue on day 1 and day 7 (Figure 3A), suggesting that ICH imposes dysfunction that affects neurons in the perilesional brain. The loss in NAA was transient in the PHE regions, as NAA was largely restored by day 14. Unexpectedly, NAA in the ATNE was still reduced 2 weeks after ICH, suggesting the more permanent nature of injury/dysfunction in the region containing no edema than in the regions spared by edema. The NAA concentration in PHE recovered from 9.70 ± 2.7 mM at day 1 to 14.79 ± 2.5 mM (*p <* 0.001) at day 14, values equivalent to NAA levels in the contralesional region. However, there was virtually no recovery in NAA content in the ATNE between day 1 and 14 (9.45 ± 3.1 vs. 9.99 ± 2.1 mM, *p* = 0.51). There was no change in NAA content in contralesional regions (13.7 ± 2.1 vs. 14.5 ± 2.0, *p* = 0.36) between the first and last time points.

As compared to the contralesional tissue, the Cr concentration in response to ICH was reduced (*p <* 0.001) within both the PHE and ATNE regions and remained reduced for 14 days, through the end of the experiment (Figure 3B). Unlike in the case of NAA, which was fully restored, Cr levels both in PHE and ATNE were only partially restored (3.62 ± 1.7 mM to 6.23 ± 2.9 mM; *p* = 0.001 for PHE and 3.46 ± 1.2 vs. 5.15 ± 2.4 mM, *p* = 0.023 for ATNE) 14 days after ICH as compared to contralesional tissue that was not affected by ICH (9.43 ± 2.2 mM vs. 10.7 ± 2.3 mM, *p* = 0.08).

Cho concentrations in the PHE and ATNE were also sharply reduced as determined on day 1 and 7 after ICH (*p <* 0.001), compared to the contralesional side (Figure 3C). The Cho levels were restored by day 14 in the PHE, but no signs of recovery were seen at this time point in ATNE. Cho concentration was unchanging in the contralesional side throughout the experiment.

There was no significant correlation between hematoma and edema volume with NAA, Cr, and Cho in all three spectroscopic voxel locations. No change was seen in the ratio of water signals between PHE, ATNE, and the contralesional side over time (*P* = NS), suggesting that the observed patterns of metabolic alterations are not due to changes in the reference tissue water content.

## Discussion

It is generally accepted that autologous blood infusion animal models provide a strong translational platform for studying the pathogenesis of ICH and testing therapeutic approaches (Wagner et al., 1999; Andaluz et al., 2002; Gu et al., 2009). The present study evaluated temporal metabolic changes in the perihematoma region using MRS in a piglet model of ICH. As compared to the rodent models of ICH, the larger brain size of piglets and thus ability to generate larger hematoma aids in the more qualitative and quantitative analyses of the changes in the perihematomal regions. Our present study compared metabolic heterogeneity in the perihematomal/ICH-adjacent tissue, sampled to capture metabolites within the edema-affected tissue (PHE) and the location devoid of edema (ATNE). We found that the concentrations of three measured metabolites (NAA, Cho and Cr) were reduced as early as day 1 after ICH in both PHE and ATNE regions. However, over the course of 14 days these metabolites were largely restored in the PHE, but not in the ATNE. These data suggest the potential of MR spectroscopy to identify salvageable tissue around the hematoma. Since perihematomal tissue demonstrating edema recovered more effectively from metabolic disturbances, our data may suggest that edema could play a homeostatic role regarding integrity of perihematomal tissue.

NAA and *N*-Acetylaspartylglutamate (NAAG) are both synthetized by neurons in the brain and upon secretion are internalized and metabolized by glial cells (Baslow, 2010). Both NAA and NAAG produce overlapping magnetic resonance spectral peaks at 2.01 ppm, not permitting differentiation between the individual metabolites. The consensus is that NAA concentration is a total (NAA ++ NAAG) concentration. Reduction of NAA concentration typically reflects some types of neuronal dysfunction or direct neuronal loss. Since ICH is known to produce extensive brain damage either through mass effect-mediated injury or through biochemical-mediated injury via toxic products of blood, blood-brain-barrier disruption, and brain edema (Aronowski and Zhao, 2011; Keep et al., 2012b; Bobinger et al., 2018), neurons surrounding the hematoma are adversely affected by ICH. Thus, it is not surprising that NAA levels in tissue close to the hematoma both in areas with and without edema show significant reduction at day 1 after ICH. However, the essential question is whether this early neuronal injury is transient and associated with reversible damage. Our results indicate that NAA gradually recovered in the PHE but not in the ATNE. Ultimately, these results suggest that neural tissue integrity, at least in the PHE region, is retained after ICH. The irreversible decline of NAA in the ATNE suggests more permanent metabolic dysfunction or neuronal loss. Our data also raise the possibility that the presence of edema could provide a protective cushioning from irreversible damage.

Creatine is a key molecule in the regulation of energy metabolism in the brain and is responsible for re-synthesis of ATP in glial cells (Rackayova et al., 2017). Here we found a significant loss of Cr levels in both the PHE and ATNE regions, suggesting either higher cellular energy demand-mediated depletion of (increased catabolism) Cr and/or its reduced synthesis. In contrast to the transient nature of NAA reduction in the PHE region, the Cr level decline was more sustained and still significantly reduced in both the PHE and ATNE regions at 14 days after ICH. However, over time a weak increasing trend of Cr concentration in both the PHE and ATNE regions suggests regional variance in metabolic dysfunction around the injury. Often, Cr levels are presumed to be constant and have been utilized as a normalizing factor in reporting metabolite concentrations. While this assumption is valid for a healthy brain, under certain pathological conditions Cr levels are altered (Burklen et al., 2006; Andres et al., 2008), including in our study. Thus, appropriate caution must be taken using Cr as a constant value under pathological conditions and reporting metabolite concentrations relative to Cr.

Unlike other pathologies where an elevated concentration of choline has been recognized as a marker of progressive pathologies (Fayed et al., 2008; Karaszewski et al., 2010), here we are reporting a significant reduction in choline in both the PHE and ATNE during the first 7 days after ICH onset. By day 14, choline is restored in the PHE but not in the ATNE region, suggesting transient and sustainable metabolic dysfunction, respectively. Choline, which is localized in all types of cells, serves as a marker for overall membrane integrity, and as such in reference to our study, suggests that the integrity of cell membranes varies around brain tissue surrounding the hematoma. Previously in ischemic stroke animal studies an inverse relation between regional cerebral blood flow (CBF) and choline concentration had been documented (Scremin and Jenden, 1991). The relation between regional metabolic variation and CBF in the ICH model will be further investigated in the future. In addition, the changes in all three metabolite concentrations over time in the PHE, as compared to the absence of changes in the contralesional hemisphere, not only validate the methodology but also suggests a hemispheric rather than global compromise in response to ICH in this model.

In our piglet ICH model, we attempted to generate a modest size hematoma that results in a modest level of edema, which did not result in midline shift or cause too severe mass effect that inflicts irreversible injury and morbidity, as it happens in many humans with large bleeds. This approach allowed for more “uncontaminated” probing of the metabolic characteristics in the perihematomal zone, specifically edema-affected brain tissue vs. perihematomal non-edematous brain tissue. Here, we unexpectedly found the more effective reversal of metabolite levels in the PHE regions as compared to the non-edematous tissue. We certainly predict that the mild edema is beneficial and not detrimental compared to larger degrees of peri-hematoma edema, which leads to compression of local microvasculature resulting in hypoperfusion and compression of white matter tract resulting in anomalous nerve conduction. Collectively, this data may suggest potential beneficial function for mild edema in re-establishing homeostasis in peri-hematoma tissue after ICH.

Our study is limited by a small sample size and will need to be reproduced in larger studies. Another limitation is the variation in edema volume among animals induces partial volume effects in the PHE voxels with normal appearing tissues. In addition, the evolution of hematoma over time also complicates voxel placement and induces partial volume effects. Validation of our findings will require additional studies including PHE tissue histology and *in vitro* metabolite concentration measurements using mass spectrometry or High Performance Liquid Chromatography (HPLC).

In conclusion, this study demonstrated the possible application of MR spectroscopy in monitoring the sub-acute phase of ICH to identify salvageable tissue in the perihematoma region. Also, reversible metabolic recovery in the PHE as compared to the tissue adjacent to the injury without edema, suggesting the potentially beneficial role of edema.

## Data Availability

The datasets generated for this study are available on request to the corresponding author.

## Ethics Statement

Animal studies were approved by our institution’s Animal Welfare Committee. The studies followed the guidelines outlined in the Guide for the Care and Use of Laboratory Animals from the National Institutes of Health.

## Author Contributions

MH designed the study, carried out MRI quantitative analysis, and drafted the manuscript. RG wrote Matlab code for spectral integration and assisted in optimizing MRI acquisition. XiZ induced hematoma and supervised the surgery. SG and SB performed regions of interest analysis and generated final plots and figures. KH provided qualitative and quantitative quality assurance of data analysis. XuZ performed statistical analysis. S-MT and GS performed animal surgery. SS supervised the study, edited manuscript, and provided necessary resources. JA is the PI of the study.

## Conflict of Interest Statement

The authors declare that the research was conducted in the absence of any commercial or financial relationships that could be construed as a potential conflict of interest.
